# Implementing an infection control and prevention program decreases the incidence of healthcare-associated infections and antibiotic resistance in a Russian neuro-ICU

**DOI:** 10.1186/s13756-018-0383-4

**Published:** 2018-07-31

**Authors:** Ksenia Ershova, Ivan Savin, Nataliya Kurdyumova, Darren Wong, Gleb Danilov, Michael Shifrin, Irina Alexandrova, Ekaterina Sokolova, Nadezhda Fursova, Vladimir Zelman, Olga Ershova

**Affiliations:** 1 Center for Data-Intensive Biotechnology and Biomedicine, Skolkovo Institute of Science and Technology, Moscow, Russia; 2 Department of Intensive Care, Burdenko National Medical Research Center of Neurosurgery, Moscow, Russia; 3 Division of Infectious Diseases, Keck School of Medicine, University of Southern California, Los Angeles, USA; 4 Laboratory of Biomedical Informatics, Burdenko National Medical Research Center of Neurosurgery, Moscow, Russia; 5 IT Department, Burdenko National Medical Research Center of Neurosurgery, Moscow, Russia; 6 Department of Microbiology, Burdenko National Medical Research Center of Neurosurgery, Moscow, Russia; 7 Federal Budget Institution of Science “State Research Center for Applied Microbiology & Biotechnology” (SRCAMB), Moscow, Russia; 8 Department of Anesthesiology, Keck School of Medicine, University of Southern California, Los Angeles, USA; 9 Department of Epidemiology and Infection Control, Burdenko National Medical Research Center of Neurosurgery, Moscow, Russia

**Keywords:** Cross infection, Intensive care unit, Infection control, Drug resistance, Survival analysis

## Abstract

**Background:**

The impact of infection prevention and control (IPC) programs in limited resource countries such as Russia are largely unknown due to a lack of reliable data. The aim of this study is to evaluate the effect of an IPC program with respect to healthcare associated infection (HAI) prevention and to define the incidence of HAIs in a Russian ICU.

**Methods:**

A pioneering IPC program was implemented in a neuro-ICU at Burdenko Neurosurgery Institute in 2010 and included hand hygiene, surveillance, contact precautions, patient isolation, and environmental cleaning measures. This prospective observational cohort study lasted from 2011 to 2016, included high-risk ICU patients, and evaluated the dynamics of incidence, etiological spectrum, and resistance profile of four types of HAIs, including subgroup analysis of device-associated infections. Survival analysis compared patients with and without HAIs.

**Results:**

We included 2038 high-risk patients. By 2016, HAI cumulative incidence decreased significantly for respiratory HAIs (36.1% vs. 24.5%, *p*-value = 0.0003), urinary-tract HAIs (29.1% vs. 21.3%, p-value = 0.0006), and healthcare-associated ventriculitis and meningitis (HAVM) (16% vs. 7.8%, p-value = 0.004). The incidence rate of EVD-related HAVM dropped from 22.2 to 13.5 cases per 1000 EVD-days. The proportion of invasive isolates of *Klebsiella pneumoniae* and *Acinetobacter baumannii* resistant to carbapenems decreased 1.7 and 2 fold, respectively. HAVM significantly impaired survival and independently increasing the probability of death by 1.43.

**Conclusions:**

The implementation of an evidence-based IPC program in a middle-income country (Russia) was highly effective in HAI prevention with meaningful reductions in antibiotic resistance.

**Electronic supplementary material:**

The online version of this article (10.1186/s13756-018-0383-4) contains supplementary material, which is available to authorized users.

## Background

Infection prevention and control (IPC) programs have been repeatedly shown to be effective at decreasing the incidence of healthcare-associated infections (HAIs). A landmark paper on this topic in 1985 showed a 32% decrease in the hospital infection rate after 5 years of an ongoing IPC program [1]. In 1999 the CDC identified seven key evidence-based elements of an effective IPC strategy including voluntary participation of all hospitals, standardized case definitions and protocols, targeted interventions for high risk patient populations, risk adjusted comparisons of infection rates across hospitals, education and adequacy of resources, and feedback to healthcare providers [2]. The elements of an IPC program have since been significantly updated, forming the concept of “multimodal strategy” [3].

To prevent HAIs, the WHO recommends implementing an IPC program in every acute healthcare facility [4]. However, according to the most-recent survey, only 29% of 133 countries surveyed have IPC programs in all tertiary hospitals [3]. In Russia, IPC programs are also not widely used. The rate of HAIs in Russia has been heavily underestimated for decades. In 2016 it was reported to be approximately 0.08% (24,771 [5] cases per 31.3 million hospitalized patients [6]) yet a concurrent meta-analysis which included Russia reported the prevalence of HAIs at 15.5% [7]. According to the latest World Bank report, Russia has a gross national income per capita of US $9720, corresponding to a middle-income country [8].

Besides significant underreporting of HAIs, Russia faces other challenges in establishing IPC programs, such as lack of commitment, punishment-based HAI reporting systems, lack of expertise, and inadequate allocation of resources [9]. Since the dissolution of the Soviet Union, Russia has made some progress in adopting the IPC programs [10]. A pioneering Russian hospital where an evidence-based IPC program was implemented in 2010 is Burdenko National Medical Research Center of Neurosurgery (NSI) in Moscow. Herein we report the results of our study which aimed to evaluate the impact of this program on HAI prevention in the ICU.

## Methods

### Study design and healthcare facility

This study was a prospective observational cohort study with annual interim data analyses. The study was done in the neuro-ICU department at NSI in Moscow, Russia. NSI is a specialized neurosurgical hospital with 300 beds that cares for approximately 8000 patients per year, 95% of whom undergo surgery. The NSI ICU has 38 single-bed rooms with a flow of approximately 3000 patients per year.

### Infection prevention and control program

**Fig. 1:**
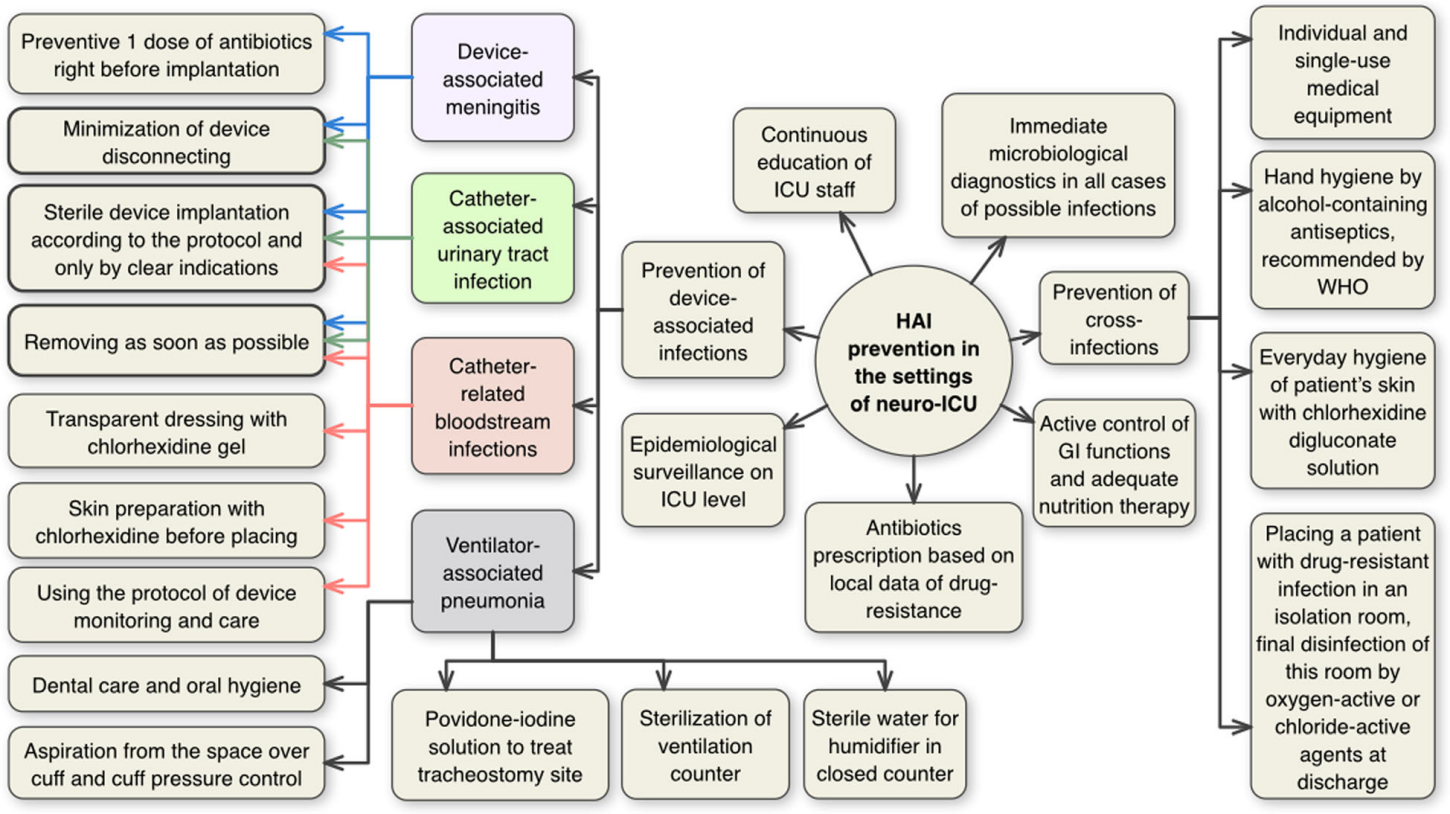
The key elements of multimodal strategy and core infection prevention and control measures in the scope of Infection Prevention and Control (IPC) Program implemented in 2010 in neuro-ICU at Burdenko National Medical Research Center of Neurosurgery in Russia

In September 2010, an IPC program was first set up in the neuro-ICU, inspired by the results of the European HELICS-ICU program [11]. The protocols for our IPC program were adopted from the 2007 CDC guidelines [12] and included three key components: education, infection prevention measures, and surveillance (Fig. 1). The surveillance software was designed in-house and integrated in the NSI electronic health record system [13]. At the time of initiation of this program, an antibiotic stewardship program was in existence at our facility. However, during the study period there were refinements to this program and coordination of antibiotic stewardship initiatives with the infection control program.

### Patients

We studied a high-risk patient population, which we defined as patients who required > 48 h of care in the neurosurgical ICU. All of these patients were qualified to participate in the study until discharge or death. Enrollment period was between January 1st, 2011 and December 31st, 2016. Following ICU discharge, the parameters of total length of stay and outcome were collected.

To identify cases of HAIs, we used the 2008 CDC definition [14]. Four types of HAIs were surveilled: bloodstream, respiratory and urinary-tract infections, and healthcare-associated ventriculitis and meningitis (HAVM). We specifically focused on the subgroup of device-related infections, such as central line-associated bloodstream infections (CLABSI), ventilator-associated pneumonia (VAP), catheter-associated urinary-tract infections (CAUTI), and external ventricular drain (EVD)-associated HAVM. In accordance with the CDC case definitions, an infection was considered device-related if the patient had a device in place for > 48 h prior to developing the HAI [12].

In addition to HAIs, we monitored superficial surgical-site infections (SSSI) after neurosurgery, and ICU-acquired intestinal dysfunction. The latter was clinically defined by the presence of one or more of the following gastrointestinal symptoms, as delineated in the literature [15]: vomiting, diarrhea, absence or abnormality of bowel sounds, bowel dilation, gastrointestinal bleeding, or increased nasogastric aspirate volume (> 500 ml/day).

### Data collection and preprocessing

Data was collected prospectively on a daily basis and incorporated 54 different characteristics (Additional file 1: Table S1). The spectrum and susceptibility profile of identified organisms causing the HAIs was built for each infection type. In January of each year, interim analysis was performed, and the results were then disseminated to NSI staff to encourage compliance with IPC measures.

### Microbiological analysis

Clinical samples were collected form patients with HAIs and delivered to the microbiological laboratory without delay. Blood and CSF samples were processed using BD BACTEC (Becton, Dickinson and Company, USA). All samples of pure bacterial cultures underwent automated identification by VITEK®2 (Biomerieux, France) with standard AST Cards. Selected samples of pure bacterial cultures were subsequently identified by MALDI-TOF MS, MALDI Biotyper® (Bruker Daltonik GmbH, Germany). Minimal inhibitory concentrations obtained from VITEK®2 were interpreted in accordance with the current CLSI guidelines [16]. A profile of antibiotic resistance for each strain was built using the WHONET software [17].

### Statistical analysis

Statistical analysis was performed in Python3.6 using StatsModels [18] and Scipy [19]. Categorical variables for dichotomous events were reported as number of events of one category with percentage and 95% confidence interval (CI) for binomial distribution. Continuous variables were reported as a median value with first and third quartiles (Q1; Q3). Incidence of HAIs was calculated as a number of cases per 100 high-risk patients or as a number of cases per 1000 patient-days. DA-HAIs were measured as cases per 1000 device-days. Device utilization ratio (DUR) was calculated as proportion of device-days to patient-days. We used Chi-square test to compare binary and categorical variables and linear regression analysis to compare continuous variables over years. In survival analysis we used Cox regression, including HAIs, diagnosis, surgeries, and preexisting characteristics. Log-rank test was used to compare survival curves. *P*-values below 0.05 were considered statistically significant.

## Results

A total of 2038 patients of all ages and both genders were included in the study during 6 years (the study data set is available at 10.5281/zenodo.1021503). The code for data analysis is available at https://github.com/KseniaErshova/IPC_paper.git.

Study population included 50% males, 16.9% children under 18 years, and a patient median age of 46 [Q1;Q3: 26.0; 59.0] years. The patients were uniformly distributed across the years by disease types, surgery types, and patient features. However, the number of lethal outcomes and the length of stay in the ICU decreased from 2011 to 2016. The baseline characteristics of the study population for each year and averaged over the 6 years are shown in Table 1.

**Table 1:** Baseline characteristics of the study population by years

	Parameters	Total	2011	2012	2013	2014	2015	2016	*p*-value
			No of pts. (%)	No of pts. (%)	No of pts. (%)	No of pts. (%)	No of pts. (%)	No of pts. (%)	
	Patients, total	2038	313 (100%)	350 (100%)	361 (100%)	341 (100%)	326 (100%)	347 (100%)	1.000
	Children	345 (16.9%)	52 (16.6%)	57 (16.3%)	58 (16.1%)	65 (19.1%)	42 (12.9%)	71 (20.5%)	0.315
	Male gender	1020 (50%)	154 (49.2%)	184 (52.6%)	186 (51.5%)	168 (49.3%)	164 (50.3%)	164 (47.3%)	0.976
Diagnosis	Brain trauma	255 (12.5%)	43 (13.7%)	54 (15.4%)	51 (14.1%)	41 (12.0%)	28 (8.6%)	38 (11.0%)	0.192
	Brain tumor	1271 (62.4%)	185 (59.1%)	221 (63.1%)	240 (66.5%)	200 (58.7%)	209 (64.1%)	216 (62.2%)	0.911
	Congenital disorders	23 (1.1%)	4 (1.3%)	5 (1.4%)	3 (0.8%)	7 (2.1%)	2 (0.6%)	2 (0.6%)	0.436
	Vascular brain diseases	454 (22.3%)	77 (24.6%)	60 (17.1%)	63 (17.5%)	89 (26.1%)	80 (24.5%)	85 (24.5%)	0.066
	Other diseases	29 (1.4%)	3 (1.0%)	10 (2.9%)	4 (1.1%)	4 (1.2%)	4 (1.2%)	4 (1.2%)	0.302
Surgeries	Craniotomy	1537 (75.4%)	230 (73.5%)	261 (74.6%)	279 (77.3%)	262 (76.8%)	245 (75.2%)	260 (74.9%)	0.998
	INSD	650 (31.9%)	101 (32.3%)	130 (37.1%)	124 (34.3%)	112 (32.8%)	94 (28.8%)	89 (25.6%)	0.227
	Endovascular surgery	194 (9.5%)	31 (9.9%)	37 (10.6%)	26 (7.2%)	40 (11.7%)	25 (7.7%)	35 (10.1%)	0.407
	EETS	87 (4.3%)	13 (4.2%)	15 (4.3%)	15 (4.2%)	14 (4.1%)	15 (4.6%)	15 (4.3%)	1.000
	Spinal surgery	4 (0.2%)	1 (0.3%)	0 (0.0%)	0 (0.0%)	0 (0.0%)	2 (0.6%)	1 (0.3%)	0.377
	Other surgeries	873 (42.8%)	151 (48.2%)	161 (46.0%)	156 (43.2%)	146 (42.8%)	127 (39.0%)	132 (38.0%)	0.523
Outcomes	Recovery	80 (3.9%)	15 (4.8%)	14 (4.0%)	14 (3.9%)	19 (5.6%)	9 (2.8%)	9 (2.6%)	0.365
	Positive dynamics	934 (45.8%)	133 (42.5%)	153 (43.7%)	170 (47.1%)	159 (46.6%)	150 (46.0%)	169 (48.7%)	0.934
	No dynamics	210 (10.3%)	34 (10.9%)	41 (11.7%)	37 (10.2%)	30 (8.8%)	29 (8.9%)	39 (11.2%)	0.818
	Negative dynamics	505 (24.8%)	81 (25.9%)	67 (19.1%)	78 (21.6%)	92 (27.0%)	96 (29.4%)	91 (26.2%)	0.153
	Death	307 (15%)	50 (16.0%)	75 (21.4%)	62 (17.2%)	41 (12.0%)	41 (12.6%)	38 (11.0%)	0.009
		Median [Q1;Q3]	Median [Q1;Q3]	Median [Q1;Q3]	Median [Q1;Q3]	Median [Q1;Q3]	Median [Q1;Q3]	Median [Q1;Q3]	*p*-value
	Age, years	46 [26.0; 59.0]	44 [25.0; 57.0]	44 [25.0; 58.0]	47 [26.0; 60.0]	44 [25.0; 57.0]	50 [30.0; 59.75]	48 [24.5; 60.5]	0.099
	CCI score	3 [2.0; 5.0]	3 [2.0; 4.0]	3 [2.0; 5.0]	3 [2.0; 5.0]	3 [2.0; 4.0]	3 [2.0; 5.0]	3 [2.0; 4.0]	1.000
	Length of stay in ICU, days	10 [6.0; 22.0]	13 [7.0; 27.0]	12 [6.0; 25.0]	10 [6.0; 24.0]	8 [6.0; 22.0]	9 [6.0; 22.0]	8 [5.0; 17.0]	0.010

Abbreviations: *INSD* Implantation of neurosurgical devices, *EETS* Endoscopic endonasal transsphenoidal surgery, *CCI* Charlson comorbidity index

### HAIs and patients’ stay in the ICU

**Fig. 2:**
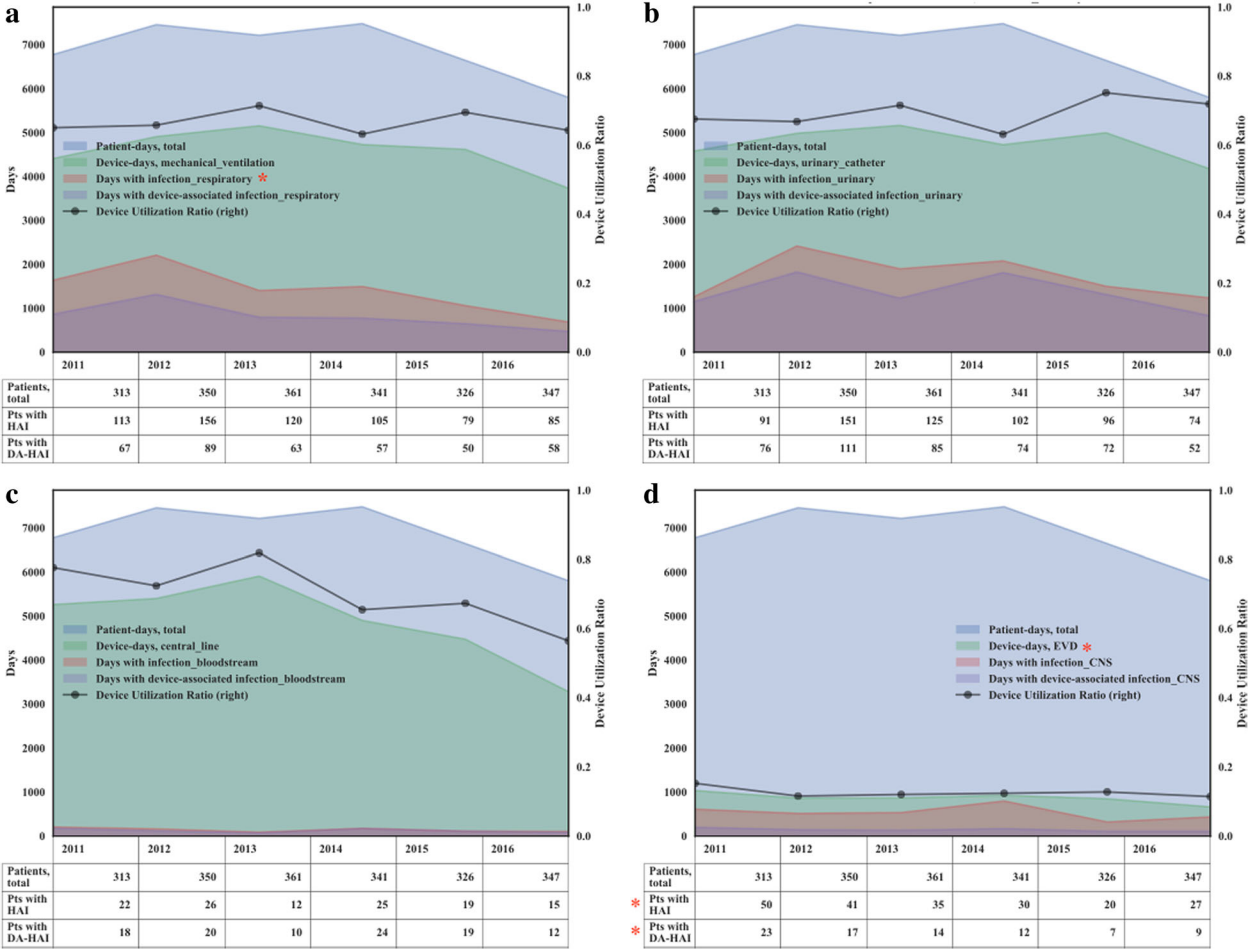
Proportion of time-dependent variables (total patient days, device days, days with infection, days with device-associated infection; unstacked area plot), number of patients, and device utilization ratio (right y-axis) for corresponding device by the years for each HAI. **a** HA respiratory infection and mechanical ventilation. **b** HA urinary tract infection and urinary catheter. **c** HA bloodstream infection and central line. **d** HA ventriculitis and meningitis and EVD. Number of patients in the study in each year is presented in a table below each graph. HA - healthcare-associated; HAI - healthcare-associated infection; DA-HAI - device-associated HAI. Star (*) shows *p*-value > 0.05 in a linear regression analysis over years

A median number of 344 [Q1;Q3: 330; 349] patients per year accounted for a median 6998 [Q1;Q3: 6678; 7399] patient-days per year (Additional file 1: Table S2). Since the number of patients increased from 2011 to 2016 by an average of 2.3% annually and the number of patient-days gradually decreased simultaneously by 2.7% per year (from 6778 to 5809), an average patient spent less time in the ICU, from the median of 13 days [Q1;Q3: 7.0; 27.0] in 2011 to 8 days [Q1;Q3: 5.0; 17.0] in 2016, *p*-value = 0.01 (Table 1). We found that over the six-year study, the lowest percentage of DA-HAIs was in the HAVM group: 40.4% [95% CI 33.6–47.1]. The highest percentage of DA-HAIs was in healthcare-associated bloodstream infections: 86.6% [95% CI 80.4–92.7]. Thus, most healthcare-associated bloodstream infections were CLABSI, whereas less than half of HAVM cases were EVD-associated (Additional file 1: Table S3, Fig. 2).

DUR was relatively high for mechanical ventilation (0.65 [Q1;Q3: 0.65; 0.69]), central line (0.70 [Q1;Q3: 0.66; 0.76]), and urinary catheter (0.70 [Q1;Q3: 0.67; 0.72]), but low for EVD (0.12 [Q1;Q3: 0.12; 0.13] (Additional file 1: Table S2, Fig. 2). Although, DURs varied slightly over time, we observed a significant decrease in the number of days with respiratory HAIs: from 1643 days in 2011 to 690 in 2016 (mean annual reduction rate 11.9%, *p*-value = 0.038), while the number of days with VAP remained unchanged (Fig. 2a). The number of patients with HAVM and with DA-HAVM decreased significantly from 2011 to 2016 (Fig. 2d).

### Incidence of healthcare-associated infections

**Fig. 3:**
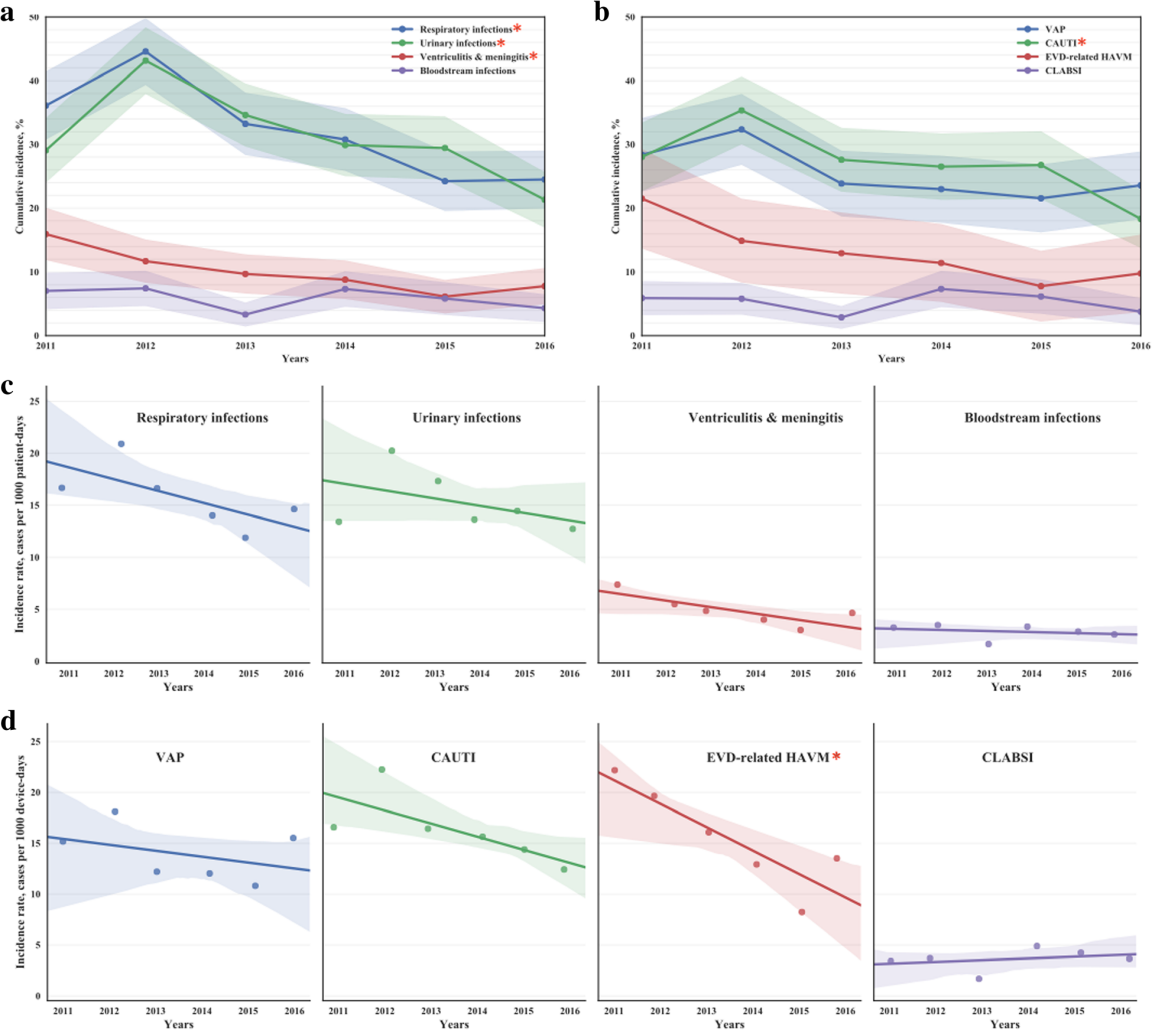
The incidence rate of HAIs in high-risk patients in 2011–2016. **a** Cumulative incidence of HAIs, cases per 100 patients in study population. **b** Cumulative incidence of device-associated HAIs per 100 patients with devices. **c** incidence rate of all HAIs, cases per 1000 patient-days. **d** incidence rate of device-associated HAIs, cases per 1000 device-days in patients with devices. Star (*) marks p-value < 0.05 in group comparison; in **a** and **b** *p*-values obtained from Chi-square test, in **c** and **d**—from linear regression. In **a** and **b** shadowed area shows 95% confidence interval, in **c** and **d**—the confidence interval for the regression estimate. Abbreviations: VAP - Ventilator-Associated Pneumonia; CLABSI - Central Line-associated Bloodstream Infection; CAUTI - Catheter-Associated Urinary Tract Infection

The incidence of all-cause HAIs and DA-HAIs was analyzed. The cumulative incidence of all-cause HAIs decreased significantly for respiratory infections (from 36.1% [95% CI 30.8–41.4] in 2011 to 24.5% [95% CI 20.0–29.0] in 2016, p-value = 0.0003), urinary tract infections (from 29.07% [95% CI 24.0–34.1] in 2011 to 21.33% [95% CI 17.0–25.6], p-value = 0.0006), and HAVM (from 15.97% [95% CI 11.9–20.0] in 2011 to 7.78% [95% CI 5.0–10.6] in 2016, p-value = 0.004) (Fig. 3a, Additional file 1: Table S4). Time-adjusted incidence rate of all-cause HAIs identified a declining trend for all four types of HAIs (Fig. 3c). In the group of DA-HAIs, only the cumulative incidence of CAUTI decreased significantly, from 28.04 [95% CI 22.7–33.4] per 100 patients with a urinary catheter in 2011 to 18.31 [95% CI 13.8–22.8] in 2016, p-value = 0.026 (Fig. 3b, Additional file 1: Table S4). However, once we adjusted incidence to the device-days at risk, EVD-associated HAVM demonstrated a significant drop from 2011 to 2016 (22.2 vs. 13.5 cases per 1000 EVD-days, respectively) (Fig. 3d, Additional file 1: Table S2). Risk-adjusted incidence of VAP and CAUTI also trended toward a decrease. The incidence rate of CLABSI did not change and remained at the median level of 3.7 [Q1;Q3: 3.5; 4.1] per 1000 central line-days (Fig. 3d, Additional file 1: Table S2). Of note, in 2012 the rates of respiratory and urinary HAIs as well as VAP and CAUTI spiked increasing 4–14% compare to 2011 (Additional file 1: Table S4). Therefore, the reduction in infection rate at the end of the study period in 2016 was more pronounced when compared to peak rates seen in 2012.

### Microbiological profile of HAIs

**Fig. 4:**
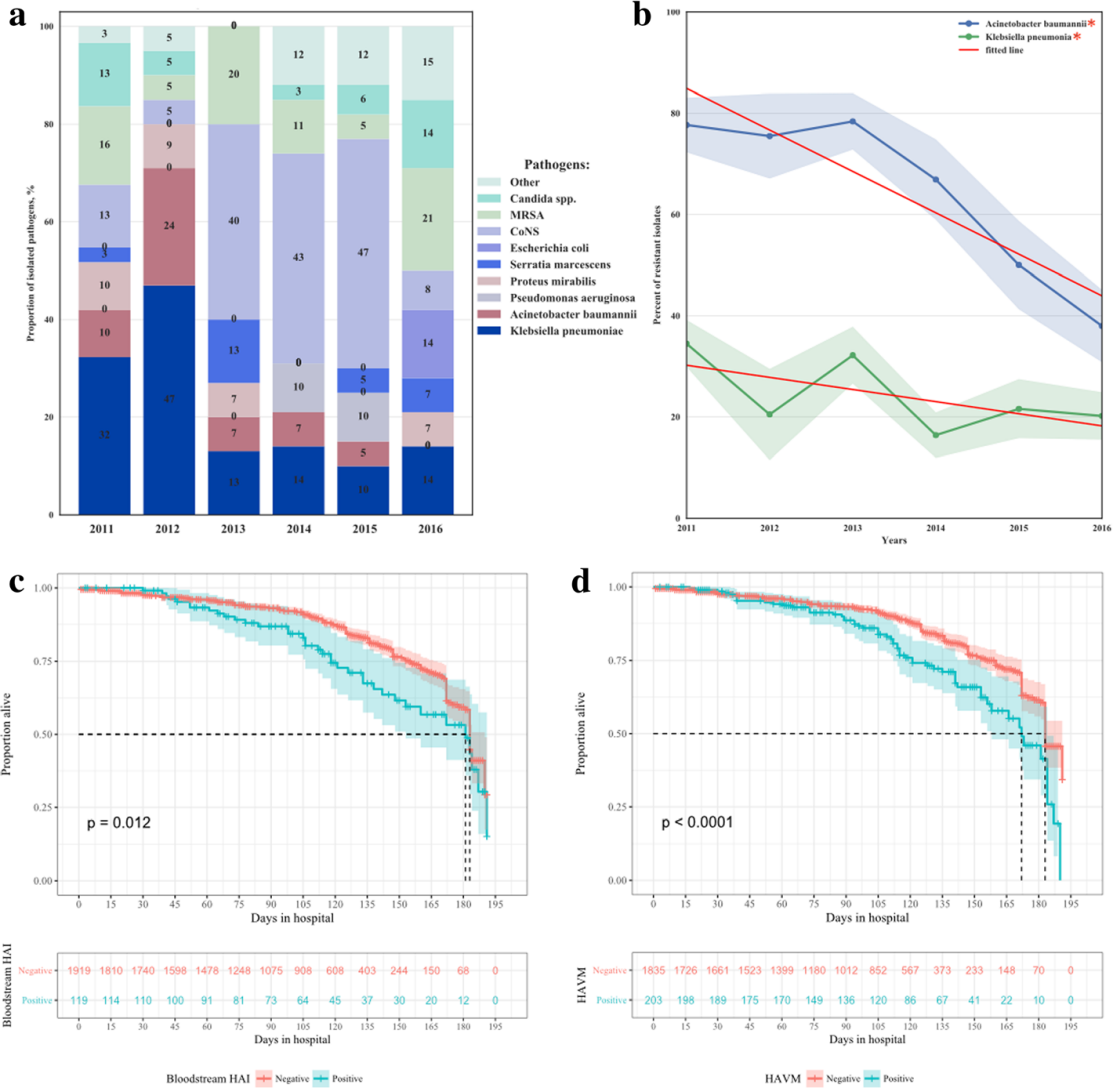
The surveillance results at the high-risk patient population in neuro-ICU from 2011 to 2016. **a** the dynamics of etiological structure of bloodstream HAIs. **b** The proportion of bacterial isolates resistant to Imipenem. **c** Survival curves for patients with and without bloodstream HAIs throughout the entire study period. **d** Survival curves for patients with and without HAVM throughout the entire study period. Shadowed areas with corresponding color at **b, c** and **d** represent 95% confidence interval. For survival curves, number of patients at risk in each group is presented in table below each graph. Abbreviations: MRSA - methicillin-resistant *Staphylococcus aureus*; CoNS - coagulase-negative Staphylococci, HAVM - healthcare-associated ventriculitis and meningitis

We observed that in 2011–2012 approximately half of bloodstream HAIs were caused by *Klebsiella pneumoniae* and *Acinetobacter baumannii.* However, in 2016 the proportion of *K. pneumoniae* decreased to 14% from a high of 47% in 2012 and *A. baumannii* did not appear on the profile for the first time (Fig. 4a). There was a tendency for Gram-negative species to be replaced by Gram-positive species (Fig. 4a). For other HAIs, the etiological spectrum remained relatively stable over time (Additional file 1: Figures S1–S3).

By 2016 *K. pneumoniae* became more susceptible to the most-tested antibiotics: there were significantly fewer isolates resistant to cephalosporins, ciprofloxacin, and imipenem as compared to 2011 (Additional file 1: Figure S4). The proportion of imipenem-resistant *K. pneumoniae* decreased from 34.5% [95% CI 29.9–39.1] in 2011 to 20.2% [95% CI 15.6–24.8], *p*-value < 0.001 (Fig. 4b). Dramatic changes were found in cephalosporin resistance, e.g. in 2011 there were 90.3% isolates resistant to cefepime [95% CI 87.4–93.1] vs. 45.6% [95% CI 39.9–51.4] in 2016, *p*-value < 0.001 (Additional file 1: Figure S4).

The number of imipenem-resistant isolates of *A. baumannii* decreased from 77.7% [95% CI 72.3–83.0] in 2011 to 38% [95% CI 30.9–45.1] in 2016, *p*-value < 0.001 (Fig. 4b). While the proportion of ampicillin/sulbactam-resistant isolates increased from 48.1% [95% CI 34.8–61.5] in 2011 to 82% [95% CI 76.2–87.9] in 2016, *p*-value < 0.001, the resistance to the rest of tested antibiotics remained virtually unchanged (Additional file 1: Figure S5). These changes in resistance occurred with a concurrent reduction in antibiotic utilization over the study period. Antibiotic use was measured as antibiotic-days per 1000 patient-days. The rate of antibiotic utilization was initially 1066 antibiotic days per 1000 patient-days in 2011. This highlights that multiple antibiotics were administered in many patients and a high overall usage rate was in effect. Over the six-year study period the utilization rate consistently declined. In 2016 the utilization rate was 807 antibiotic days per 1000 patient-days.

### Survival analysis in patients with HAIs

Bloodstream HAIs and HAVM significantly impair survival (log-rank *p*-values = 0.012 and < 0.0001, respectively), Fig. 4c and 4d. In order to confirm their influence on mortality, multifactorial survival analyses were done by Cox regression (Additional file 1: Table S5). We confirmed that only HAVM affected survival independently from other factors, increasing the probability of death 1.43 times (95% CI 1.03–1.98, p-value = 0.034). Other types of HAIs did not influence survival (Additional file 1: Figure S6). Besides HAIs, other factors were shown to independently affect survival. While brain tumor (HR = 1.57 [95% CI 1.1–2.24], p-value = 0.012) and implantation of neurosurgical devices (HR = 1.59 [95% CI 1.24–2.03], p-value = 0.0002) enhanced mortality, craniotomy decreased mortality: HR = 0.64 [95% CI 0.48–0.87], p-value = 0.0037 (Additional file 1: Figure S7).

### ICU-acquired intestinal dysfunction

The cumulative incidence of overall intestinal dysfunctions dropped from 54.9% [95% CI 49.4–60.5] in 2011 to 23.9% [95% CI 19.4–28.4] in 2016, p-value < 0.001 (Additional file 1: Figure S8A, Additional file 1: Table S6). Intestinal dysfunction impaired survival independently increasing the probability of death 1.46 times [95% CI 1.11–1.93], p-value = 0.0069; log-rank test p-value = 0.019 (Additional file 1: Figure S8D).

## Discussion

A comprehensive IPC program with a focus on hand hygiene and patient isolation was started in NSI’s ICU in 2010 (Fig. 1a). By that time, the use of our IPC program to prevent HAIs in the ICU became a paradigm-shifting solution across Russia as HAI prevention strategies had previously remained unchanged for years and had become outdated [20].

The importance of HAI prevention programs is clearly indicated by the observation that HAIs directly deteriorate patient survival. It was found that HAIs increased the probability of death by 1.4–1.5 [21] and odds of mortality increased 1.5 to 1.9-fold [22]. In our study, we found that HAVM decreased the probability of survival by 1.43, while other HAIs did not significantly influence survival. It has been previously reported that HAVM increased mortality rate approximately three times [23]. Although the exact mechanism is not yet understood, prospective studies have found that in ICU patients, gastrointestinal dysfunction is also an independent risk factor for increased mortality [15]. We can postulate that the intestinal microbiome serves an important role in immune function and consequently, is a well described reservoir for antibiotic resistance [24]. Additionally, in critically ill patients intestinal dysbiosis could be postulated as a potential contributor to gut translocation of pathogens and may play a role in enteric absorption. In our study, ICU-acquired intestinal dysfunction decreased the probability of survival by 1.46, which is consistent with earlier studies. The implementation of IPC initiatives and the accompanying reduction in the incidence of infections, thereby reducing the requirement for antibiotics, can be assumed to at least in part account for reduction in gastrointestinal dysbiosis. This finding further highlights the potential unseen morbidity impact of IPC beyond simple measures of antibiotic utilization and resistance rates.

The implementation of the IPC program was followed by significant reduction of HAIs in the ICU. In fact, the impact of this program may actually be under-estimated. Our IPC program was implemented in 9/2010 whereas study data collection began 1/2011. Therefore, although adherence to IPC protocols would be expected to improve with greater time and familiarity, the totality of impact of this program may be under-estimated. Key initiatives, such as early removal of indwelling catheters, would be expected to have an immediate impact in the reduction of nosocomial infections. Even discounting the IPC impact in the initial months after implementation, the fact that a sustained and continued reduction in HAI rate occurred is both meaningful and serves as a reinforcement of overall utility. In high-risk ICU patients we observed a substantial decrease in HAI incidence: cumulative incidence of respiratory HAIs declined by 1.47 (from 36.1 to 24.5%), urinary tract HAIs by 1.4-fold (from 29.1 to 21.3%), HAVM by twofold (from 16 to 7.8%), CAUTI by 1.93 (from 35.4 to 18.3%) (Fig. 3), and ICU-acquired intestinal dysfunction by 2.3 fold. These results are consistent with previously reported evidence, demonstrating a reduction of HAI prevalence by approximately 1.7 fold (from 11.7 to 6.8%) [25].

We also found that the risk-adjusted incidence of EVD-related HAVM reduced 1.64 fold (from 22.2 to 13.5 cases per 1000 EVD-days) over the six-year study period. The impact of an IPC program on decreasing DA-HAI incidence has been previously reported. For example, one publication reported a 2.7-fold decrease in CAUTI episodes per 100 patients within a year after IPC implementation [26]. However, for some HAIs, like HAVM, such statistics are absent. In addition, the changes in the incidence of intestinal dysfunction could be confounded by the implementation of an advanced nutritional protocol in 2012 at the ICU.

We did find that in 2012 the rates of several infection subcategories did increase in comparison to 2011. The rate of respiratory and urinary HAIs had increases ranging from 4 to 14% compared to 2011. The reason for this increase is unclear, but we postulate that this may be related to several factors. One contributor may be that staff were educated on the appropriate identification of HAIs and utilized clear standardized case definitions. As staff became more familiar with these definitions, they may have been able to better identify cases leading to an apparent increase in infection rates. Additionally, during initial implementation of IPC protocols, staff underwent in-service training and consequently there was a specific focus on the strict adherence to protocols. However, adherence to infection control practices may wane with time, and that probably what happened in 2012. Therefore, continued reinforcement of best practices along with feedback to healthcare teams is necessary for sustained adherence to IPC initiatives. Following the re-education of staff, a renewed attention to IPC may have contributed to reductions seen in 2013 HAI rate.

Additionally, both the length of patients’ stays in the ICU and the incidence of patient mortality did decrease over the study period. Although a direct causality cannot be determined, it would be fair to postulate that the associated decrease in HAI incidence may at least have been a partial contributor for this reduction. Thus, a reduction in the rate of HAIs may result in a meaningful reduction in healthcare cost, and potential benefit in overall mortality. However, we did not monitor all other parameters that could have influenced the mortality and the length of stay, thus other explanations should be investigated. Additionally, we admit that the overall approach in patient treatment did not change much, and the DUR did not change for any of the devices we monitored.

The prevention of the spread of carbapenem-resistant, Gram-negative bacteria was named the first priority of IPC efforts by the latest WHO guidelines because these strains pose significant threat to global health [27]. We found firstly that the proportion of such Gram-negatives as *K. pneumoniae* and *A. baumannii* in the spectrum of bloodstream HAIs decreased and secondly that the resistance of both pathogens to carbapenems was significantly reduced. In our study the initial percentage of isolates resistant to imipenem was 34.5% for *K. pneumonia* and 77.7% for *A. baumannii.* By the end of the study, the percentage decreased 1.7- and 2-fold, respectively (Fig. 4b). The initial prevalence of carbapenem-resistant isolates in the NSI neuro-ICU was shown to be higher than the mean prevalence in Europe (8.1% for *K. pneumoniae* and 50% for *A. baumannii*), and in the U.S. (7.9% for *K. pneumoniae* and 49.5% for *A. baumannii*) [27]. This finding could partly be explained by the study population because we analyzed only intensive care unit patients which may be a higher risk population. However, we postulate these initial rates of carbapenem resistance were at least in part due to nosocomial cross-infection of patients.

Our hypothesis is that the implementation of IPC protocols acted in a two-fold manner with an initial reduction in nosocomial patient-to-patient transmission which consequently lead to a reduction in nosocomial infection rate. Our most critical interventions involved implementation of contact precautions utilizing gloves, gown, and mask, isolation of patients identified with carbapenemase resistance genes, and cohorting of patients with *Acinetobacter* or *Klebsiella* (Fig. 1). These efforts were paired with intensive environmental disinfection measures, skin antisepsis for indwelling devices, as well as initiates focused on hand hygiene as a multi-modal strategy (Fig. 1).

Of note, hand hygiene compliance was particularly difficult to implement with a compliance rate of 27% in 2011. Compliance with hand hygiene in the subsequent years 2012 through 2016 were 40, 69, 63, 68, and 81% respectively. The reduction in infection rate over time could reasonably be postulated to result in a secondary reduction in the necessity of broad spectrum antibiotic therapy. This reduction in antibiotic utilization is underscored by the dramatic decline in the rate of antibiotic utilization over the study period. It must be noted that an antibiotic stewardship program was in existence prior to IPC implementation. Antibiotic stewardship involved institutional protocols for perioperative antibiotic prophylaxis and for empiric antibiotic therapy. However, integration of IPC protocols, including surveillance measures may have enhanced the effectiveness of antibiotic stewardship interventions. The ultimate result was that within the study period, our observed resistance rates decreased to the level of global and regional estimations.

This improvement in susceptibility rates, is in contrast to the global trend of increasing carbapenem resistance over the past decade [27], indicating that in limited-resource settings IPC programs can be highly effective. The programs may be especially significant in healthcare settings with high levels of resistance where they can serve as a cost-effective intervention leading to a substantial clinical impact. The substantial diminution in carbapenem resistance supports the notion that implemented IPC strategies contain effective measures to prevent and control the resistance to carbapenems (Fig. 1). Moreover, this is supported by the recent WHO guidelines which affirmed that the core components of multimodal IPC strategy can help to prevent carbapenem resistance.

This paper reports a prospective study of the impact of an infection control program in a high acuity limited resource setting with regard to the reduction in HAI risk. Such studies are limited to date but have been identified by the WHO as particularly needed [27]. Thus, this study can help to fill this research gap providing insight regarding an approach to implantation of these programs and highlighting the most essential IPC components. Our results suggest that a focus on robust surveillance paired with isolation/infection control measures can promote a sustainable and meaningful reduction in HAI incidence and antibiotic resistance.

The current study has certain limitations. It is a single-center study in a highly specialized ICU facility. Thus, one should be careful when generalizing these results to other hospitals and other wards. In addition, we only studied a cohort of high-risk patients, those staying in the dedicated neuro-ICU for > 48 h—not the entire ICU population. Thus, reported HAI incidences are higher than those calculated for the entire ICU population. However, the underlying principles of our IPC program leading to the reduction of CAUTI, CLABSI, and VAP would be expected to be generalizable to other hospitalized settings with a similar expected impact.

One aspect that was not able to be fully evaluated were *Clostridium difficile* infections (CDI). The prevalence of CDI, identified by a positive PCR stool assay and compatible symptoms, was measured quarterly. However, the quarterly rate included all patients in the ICU at the time of a positive diagnosis and included patients that did not meet the defined criteria for high-risk population that were studied. Additionally, the incidence rate was low throughout the six-year period with a peak rate of 1.5% in 2011 and a nadir of 0.9% in 2015. Notably, patients who were transferred out of the ICU and subsequently developed CDI would not have been identified. Therefore, we can postulate that IPC initiatives may result in a reduction in CDI as the rate did decline from 2011; however, the low overall incidence of CDI and aforementioned limitations do not allow for definitive conclusions.

By design, the study did not include a control group (i.e. a group treated in the ICU before the IPC program had been implemented), because HAI rates without surveillance are unknown. Moreover, the decrease of HAI incidence and length of stay in the ICU could be explained by modification of clinical practices and by regression to the mean. It should be mentioned that survival analysis in our study suffers from immortal time bias. Patients in the HAI group are “immortal” until they get the infection, that favors the HAI group by lowering mortality rate in this group. Thus, HAIs have a stronger influence on survival, posing a higher risk of death in patients once they get HAIs.

## Conclusion

Implementation of an evidence-based IPC program was strongly associated with a significant reduction in HAIs in the neuro-ICU. Over a six-year period, there was a decreasing HAI incidence, reduction in the prevalence of carbapenem-resistant invasive bacterial isolates, and consequently improved patient outcomes. Our study supports the finding that an IPC program can be highly effective in a middle-income country (Russia) despite the lack of a national surveillance system and limited resources. Expansion of IPC initiatives, potentially paired with a robust antimicrobial stewardship program, should be considered in resource limited settings as a feasible cost-effective opportunity to achieve meaningful reductions in antibiotic resistance and HAI incidence.

## Additional file

Additional file 1: Supplementary materials for the research study report "Implementing an infection control and prevention program decreases the incidence of healthcare-associated infections and antibiotic resistance in a Russian neuro-ICU". (PDF 35444 kb)

## Abbreviations

CAUTI: Catheter-associated urinary tract infection
CDC: Centers for Disease Control and Prevention
CI: Confidence interval
CLABSI: Central line-associated bloodstream infections
DA-HAI: Device-associated HAI
DUR: Device utilization ratio
EVD: External ventricular drain
HAI: Healthcare-associated infection
HAVM: Healthcare-associated ventriculitis and meningitis
HR: Hazard ratio
ICU: Intensive care unit
IPC: Infection prevention and control
NSI: Burdenko National Medical Research Center of Neurosurgery
Q1; Q3: First and third quartiles
SSSI: Superficial surgical site infection
VAP: Ventilator-associated pneumonia
WHO: World Health Organization
