# Supplementary material for: Implementing an infection control and prevention program decreases the incidence of healthcare-associated infections and antibiotic resistance in a Russian neuro-ICU
Source: Antimicrob Resist Infect Control. 2018 Jul 31;7:94. doi: 10.1186/s13756-018-0383-4 (PMC6069828; doi:10.1186/s13756-018-0383-4)
Supplement: Supplementary file 1 — Supplementary materials for the research study report "Implementing an infection control and prevention program decreases the incidence of healthcare-associated infections and antibiotic resistance in a Russian neuro-ICU". (PDF 35444 kb) [file 13756_2018_383_MOESM1_ESM.pdf]

Supplementary Materials

Table 1 Supplementary. The description of parameters, collected in the daily epidemiological surveillance in the neuro-ICU department at Neurosurgical Institute, Moscow, Russia.

Group	#	The list of parameters	Values/comments
**General features**	1	Age	Age in years
	2	Gender	Male or Female
	3	Clinical diagnosis	Tumor; Trauma; Vascular diseases; Congenital disorders; Spinal diseases; Other
	4	Charlson Comorbidity Index (CCI)	0-37 points
	5	Patient's ID	Unique ID number, format XXXX/XX
**Technical parameters**	1	Day in the ICU	Length of stay in the ICU
	2	Date	Actual date, format dd/mm/yyyy
**Indwelling medical invasive devices**	1	External ventricular drain (EVD)	Yes - 1; No - 0
	2	Intracranial pressure monitoring (ICPM)	Yes - 1; No - 0
	3	Endotracheal intubation tube	Encoding:<br>0: No tube<br>1: Oral intubation tube<br>2: Nasal intubation tube<br>3: Tracheostomy tube
	4	Central line	Yes - 1; No - 0
	5	Arterial line	Yes - 1; No - 0
	6	Urinary catheter	Yes - 1; No - 0
	7	Feeding tube	Yes - 1; No - 0
	8	Pleural drainage	Yes - 1; No - 0
**The signs of disease severity**	1	Mechanical ventilation	Yes - 1; No - 0
	2	FiO_2	O_2 concentration; if on room air FiO_2=0.21
	3	Induced hypothermia	Yes - 1; No - 0
	4	Hemodialysis	Yes - 1; No - 0
	5	Total parenteral feeding	Yes - 1; No - 0
**The signs of infections**	1	Number of tracheal sanations per day	Number of tracheal sanations per day
	2	Purulent sputum	Yes - 1; No - 0
	3	Infiltration on the chest X-ray	Yes - 1; No - 0
	4	Intestinal dysfunction	Yes - 1; No - 0
	5	Body temperature	Temperature in Celsius degrees
**Surgical procedures**	1	Surgical procedure, type Craniotomy	Number of procedures
	2	Length of the surgical procedure, type Craniotomy	Length in minutes
	3	Surgical procedure, type Implantation of Neurosurgical Device (INSD)	Number of procedures
	4	Length of the surgical procedure, type INSD	Length in minutes

Supplementary Materials

Table 1 Supplementary. Continue

Group	#	The list of parameters	Values
**Surgical procedures**	5	Surgical procedure, type Spinal Surgery	Number of procedures
	6	Length of the surgical procedure, type Spinal Surgery	Length in minutes
	7	Surgical procedure, type Endoscopic Endonasal Transsphenoidal Surgery (EETS)	Number of procedures
	8	Length of the surgical procedure, type EETS	Length in minutes
	9	Surgical procedure, type Other Non-Cranial Surgeries	Number of procedures
	10	Length of the surgical procedure, type Other Non-Cranial Surgeries	Length in minutes
**Neurological & neurosurgical status**	1	Disorders of consciousness with a match to Glasgow Coma Scale (GCS)	Encoding:<br>7: full consciousness (GCS = 15),<br>6: drowsiness (GCS = 12-14),<br>5: stupor (GCS = 9-12),<br>4: coma-1 (GCS = 7-8),<br>3: coma-2 (GCS = 5-6),<br>2: coma-3 (GCS = 3-4),<br>1: persistent vegetative state
	2	RASS scale score	Points from -3 to 3
	3	Convulsions	Yes - 1; No - 0
	4	PBSS score	6-12 points
	5	CSF leakage from surgical site (CSFL-SS)	Yes - 1; No - 0
	6	CSF leakage from nose and/or ears (CSFL-NE)	Yes - 1; No - 0
**Medications**	1	Antibiotics	Number of antibiotics per day
	2	Anxiolytics	Yes - 1; No - 0
	3	Vasopressors	Yes - 1; No - 0
	4	Sedative	Yes - 1; No - 0
**Healthcare-associated infections (HAIs)**	1	Respiratory infections	Yes - 1; No - 0
	2	Bloodstream infections	Yes - 1; No - 0
	3	Intracranial CNS infections	Yes - 1; No - 0
	4	Superficial surgical site infection (SSSI)	Yes - 1; No - 0
	5	Urinary tract infections	Yes - 1; No - 0
	6	Other infections	Yes - 1; No - 0
**Overall**	**54**		

Supplementary Materials

Table 2 Supplementary. Summary of observations by years grouped by type of HAI. Mean growth rate per year is calculated as average of annual growth coefficient; p-value obtained from linear regression. Abbreviations: VAP - Ventilator-Associated Pneumonia; CLABSI - Central Line-associated Bloodstream Infection; CAUTI - Catheter-Associated Urinary Tract Infection; HAVM - Healthcare-Associated Ventriculitis & Meningitis, Q1;Q3 - first and third quartiles.

Infection	Parameter	2011	2012	2013	2014	2015	2016	Median [Q1;Q3]	Mean growth rate per year (%)	p-value
	**Patients, total**	313	350	361	341	326	347	344 [330; 349]	2.3	0.6476
	**Patient-days, total**	6778	7460	7217	7483	6644	5809	6998 [6678; 7399]	-2.7	0.2174
All HA respiratory infections	**Patients with infection**	113	156	120	105	79	85	109 [90; 118]	-2.9	0.0893
	**Days with infection**	1643	2215	1407	1499	1063	690	1453 [1149; 1607]	-11.9	0.0378
	**Incidence per 1000 patient-days**	16.7	20.9	16.6	14.0	11.9	14.6	15.6 [14.2; 16.7]	-0.6	0.1274
All HA urinary infections	**Patients with infection**	91	151	125	102	96	74	99 [92; 119]	0.3	0.2763
	**Days with infection**	1263	2424	1901	2083	1505	1239	1703 [1324; 2038]	6.9	0.5621
	**Incidence per 1000 patient-days**	13.4	20.2	17.3	13.6	14.5	12.7	14.0 [13.5; 16.6]	1.8	0.3684
All types of HAVM	**Patients with infection**	50	41	35	30	20	27	33 [28; 40]	-9.1	0.0096
	**Days with infection**	613	516	536	799	322	437	526 [457; 594]	2.6	0.4373
	**Incidence per 1000 patient-days**	7.4	5.5	4.9	4.0	3.0	4.7	4.8 [4.2; 5.3]	-5.0	0.0595
All HA bloodstream infections	**Patients with infection**	22	26	12	25	19	15	21 [16; 24]	5.5	0.4157
	**Days with infection**	208	166	91	178	114	107	140 [109; 175]	-2.4	0.1540
	**Incidence per 1000 patient-days**	3	3	2	3	3	3	3 [3; 3]	6.4	0.5900
VAP	**Patients with device**	236	275	264	248	232	246	247 [239; 260]	1.2	0.5529
	**Device-days**	4413	4912	5160	4733	4623	3738	4678 [4466; 4867]	-2.7	0.3036
	**Patients with device-associated infection**	67	89	63	57	50	58	61 [57; 66]	-0.4	0.1508
	**Days with device-associated infection**	865	1318	796	777	649	473	787 [681; 848]	-6.6	0.0849
	**Incidence per 1000 device-days**	15.2	18.1	12.2	12.0	10.8	15.5	13.7 [12.1; 15.4]	3.7	0.4373
	**Device utilization ratio**	0.65	0.66	0.71	0.63	0.70	0.64	0.65 [0.65; 0.69]	0.1	0.9793
CAUTI	**Patients with device**	271	314	308	279	269	284	282 [273; 302]	1.3	0.5948
	**Device-days**	4585	4989	5169	4729	5000	4182	4859 [4621; 4997]	-1.3	0.4815
	**Patients with device-associated infection**	76	111	85	74	72	52	75 [73; 83]	-4.2	0.1327
	**Days with device-associated infection**	1155	1830	1230	1815	1317	834	1274 [1174; 1691]	1.8	0.4969
	**Incidence per 1000 device-days**	16.6	22.3	16.4	15.7	14.4	12.4	16.0 [14.7; 16.5]	-3.7	0.0995
	**Device utilization ratio**	0.68	0.67	0.72	0.63	0.75	0.72	0.70 [0.67; 0.72]	1.8	0.3420
EVD-associated HAVM	**Patients with device**	107	114	108	105	90	92	106 [95; 108]	-2.7	0.0363
	**Device-days**	1037	865	870	928	850	665	868 [854; 914]	-7.9	0.0491
	**Patients with device-associated infection**	23	17	14	12	7	9	13 [10; 16]	-14.2	0.0049
	**Days with device-associated infection**	200	147	133	170	109	109	140 [115; 164]	-8.8	0.0585
	**Incidence per 1000 device-days**	22.2	19.7	16.1	12.9	8.2	13.5	14.8 [13.1; 18.8]	-4.2	0.0277
	**Device utilization ratio**	0.15	0.12	0.12	0.12	0.13	0.11	0.12 [0.12; 0.13]	-4.9	0.2286
CLABSI	**Patients with device**	305	345	349	327	309	318	323 [311; 341]	1.1	0.7194
	**Device-days**	5266	5403	5911	4903	4477	3283	5085 [4584; 5369]	-8.1	0.0564
	**Patients with device-associated infection**	18	20	10	24	19	12	19 [14; 20]	8.7	0.7125
	**Days with device-associated infection**	181	123	73	174	114	85	119 [92; 161]	1.1	0.3283
	**Incidence per 1000 device-days**	3.4	3.7	1.7	4.9	4.2	3.7	3.7 [3.5; 4.1]	23.2	0.5640
	**Device utilization ratio**	0.78	0.72	0.82	0.66	0.67	0.57	0.70 [0.66; 0.76]	-5.4	0.0528

Supplementary Materials

Table 3 Supplementary. Proportion of patients and days in groups device-associated HAIs (DA-HAI) vs. all HAIs different types of HAIs. P-values obtained from Chi-square test.

Infection	Parameter	Total % [95% CI]	2011 %	2012 %	2013 %	2014 %	2015 %	2016 %	Mean growth rate per year (%)	p-value
Respiratory infections	**Proportion of patients in groups DA-HAI / HAI**	58.4% [54.6-62.1]	59.3	57.1	52.5	54.3	63.3	68.2	3.2	0.88355
	**Proportion of days in groups DA-HAI / HAI**	57.3% [56.2-58.3]	52.6	59.5	56.6	51.8	61.1	68.6	6.0	0.00076
Urinary infections	**Proportion of patients in groups DA-HAI / HAI**	73.6% [70.1-77.0]	83.5	73.5	68.0	72.5	75.0	70.3	-3.1	0.95685
	**Proportion of days in groups DA-HAI / HAI**	78.6% [77.8-79.3]	91.4	75.5	64.7	87.1	87.5	67.3	-3.9	0.00000
HAVM	**Proportion of patients in groups DA-HAI / HAI**	40.4% [33.6-47.1]	46.0	41.5	40.0	40.0	35.0	33.3	-6.1	0.98644
	**Proportion of days in groups DA-HAI / HAI**	26.9% [25.4-28.5]	32.6	28.5	24.8	21.3	33.9	24.9	-1.4	0.00172
Bloodstream infections	**Proportion of patients in groups DA-HAI / HAI**	86.6% [80.4-92.7]	81.8	76.9	83.3	96.0	100.0	80.0	0.3	0.98942
	**Proportion of days in groups DA-HAI / HAI**	86.8% [84.5-89.1]	87.0	74.1	80.2	97.8	100.0	79.4	-0.6	0.44259

Table 4 Supplementary. Cumulative incidence of different types of HAIs. P-values obtained from Chi-square test. Abbreviations: VAP - Ventilator-Associated Pneumonia; CLABSI - Central Line-associated Bloodstream Infection; CAUTI - Catheter-Associated Urinary Tract Infection; HAVM - Healthcare-Associated Ventriculitis & Meningitis.

	Infection	2011	2012	2013	2014	2015	2016	p-value
Cumulative incidence (% and 95 CI), all HAIs	**Respiratory infections**	36.1% [30.8-41.4]	44.57% [39.4-49.8]	33.24% [28.4-38.1]	30.79% [25.9-35.7]	24.23% [19.6-28.9]	24.5% [20.0-29.0]	0.0003
	**Urinary-tract infections**	29.07% [24.0-34.1]	43.14% [38.0-48.3]	34.63% [29.7-39.5]	29.91% [25.1-34.8]	29.45% [24.5-34.4]	21.33% [17.0-25.6]	0.0006
	**HAVM**	15.97% [11.9-20.0]	11.71% [8.3-15.1]	9.7% [6.6-12.7]	8.8% [5.8-11.8]	6.13% [3.5-8.7]	7.78% [5.0-10.6]	0.0040
	**Bloodstream infections**	7.03% [4.2-9.9]	7.43% [4.7-10.2]	3.32% [1.5-5.2]	7.33% [4.6-10.1]	5.83% [3.3-8.4]	4.32% [2.2-6.5]	0.1343
Cumulative incidence (% and 95 CI), device-associated HAIs	**VAP**	28.39% [22.6-34.1]	32.36% [26.8-37.9]	23.86% [18.7-29.0]	22.98% [17.7-28.2]	21.55% [16.3-26.8]	23.58% [18.3-28.9]	0.2518
	**CAUTI**	28.04% [22.7-33.4]	35.35% [30.1-40.6]	27.6% [22.6-32.6]	26.52% [21.3-31.7]	26.77% [21.5-32.1]	18.31% [13.8-22.8]	0.0259
	**EVD-associated HAVM**	21.5% [13.7-29.3]	14.91% [8.4-21.5]	12.96% [6.6-19.3]	11.43% [5.3-17.5]	7.78% [2.2-13.3]	9.78% [3.7-15.9]	0.1805
	**CLABSI**	5.9% [3.3-8.5]	5.8% [3.3-8.3]	2.87% [1.1-4.6]	7.34% [4.5-10.2]	6.15% [3.5-8.8]	3.77% [1.7-5.9]	0.1399

Supplementary Materials

Table 5 Supplementary. Multifactorial Cox regression analysis of survival. Adjusted hazard ratio (HR) with 95% confidence intervals (95% CI) and p-values are presented. Red color highlights the factor increasing the likelihood of death; blue color - factors which decrease the likelihood of death. Abbreviations: SSSI - superficial surgical site infection; INSD - implantation of neurosurgical devices; EETS - endoscopic endonasal transsphenoidal surgery.

	Factor	Adjusted HR [95% CI]	p-value
General	**Age**	1.01 [1-1.01]	0.0353
	**Male gender**	0.84 [0.67-1.06]	0.1443
	**CCI**	1.04 [0.99-1.1]	0.0967
HAIs	**HAVM**	1.43 [1.03-1.98]	0.0338
	**Bloodstream HAI**	1.32 [0.91-1.92]	0.1493
	**Respiratory HAI**	1.04 [0.8-1.35]	0.7690
	**Urinary HAI**	0.56 [0.43-0.74]	0.0004
	**SSSI**	1.26 [0.8-2]	0.3192
	**Other HAIs**	0.94 [0.68-1.31]	0.7291
	**Intestinal dysfunction**	1.46 [1.11-1.93]	0.0069
Diagnosis	**Congenital disorder**	1.61 [0.49-5.31]	0.4343
	**Other diseases**	0.47 [0.06-3.41]	0.4524
	**Brain trauma**	0.84 [0.48-1.47]	0.5318
	**Brain tumor**	1.57 [1.1-2.24]	0.0122
Surgery	**Craniotomy**	0.64 [0.48-0.87]	0.0037
	**EETS**	1.02 [0.64-1.64]	0.9276
	**Endovascular surgery**	1.11 [0.75-1.66]	0.5989
	**INSD**	1.59 [1.24-2.03]	0.0002
	**Other surgeries**	0.93 [0.72-1.21]	0.5902

Supplementary Materials

Table 6 Supplementary. Summary of annual observations, intestinal dysfunction and feeding tube-associated intestinal dysfunction. Mean growth rate per year is calculated as average of annual growth coefficient; p-value obtained from linear regression, or from Chi-squared test (for cumulative incidence).

Infection	Parameter	2011	2012	2013	2014	2015	2016	Median [Q1;Q3]	Mean growth rate per year (%)	p-value
	**Patients, total**	313	350	361	341	326	347	344 [330; 349]	2.3	0.6476
	**Patient-days, total**	6778	7460	7217	7483	6644	5809	6998 [6678; 7399]	-2.7	0.2174
All-cause intestinal dysfunction	**Patients with intestinal_dysfunction**	172	178	145	125	105	83	135 [110; 165]	-13.2	0.0009
	**Days with intestinal dysfunction**	1499	1644	1274	990	970	738	1132 [975; 1443]	-12.2	0.0050
	**Cumulative incidence, % [95% CI]**	54.9 [49.4-60.5]	50.9 [45.6-56.1]	40.2 [35.1-45.2]	36.7 [31.5-41.8]	32.2 [27.1-37.3]	23.9 [19.4-28.4]	NA	NA	0.0000001
	**Incidence per 1000 patient-days**	25.4	23.9	20.1	16.7	15.8	14.3	18 [16; 23]	-10.7	0.0006
Feeding tube-associated intestinal dysfunction	**Patients with feeding tube**	273	305	308	292	273	290	291 [277; 302]	1.4	0.8565
	**Patients with device-associated intestinal dysfunction**	96	103	99	84	70	61	90 [74; 98]	-8.3	0.0118
	**Days with device-associated intestinal dysfunction**	855	983	956	555	630	625	743 [626; 931]	-3.4	0.0873
	**Device-days, feeding tube**	5269	5838	5992	5562	5430	4387	5496 [5309; 5769]	-3.1	0.2367
	**Cumulative incidence, % [95% CI]**	35.2 [29.5-40.8]	33.8 [28.5-39.1]	32.1 [26.9-37.4]	28.8 [23.6-33.9]	25.6 [20.5-30.8]	21.0 [16.3-25.7]	NA	NA	0.0490
	**Incidence per 1000 device-days**	18.2	17.6	16.5	15.1	12.9	13.9	16 [14; 17]	-5.0	0.0047
	**Proportion of patients in groups feeding tube-associated dysfunction / all-causes dysfunction**	55.8	57.9	68.3	67.2	66.7	73.5	67 [60; 68]	5.9	0.0132
	**Proportion of days in groups feeding tube-associated dysfunction / all-causes dysfunction**	57.0	59.8	75.0	56.1	64.9	84.7	62 [58; 73]	10.3	0.1782

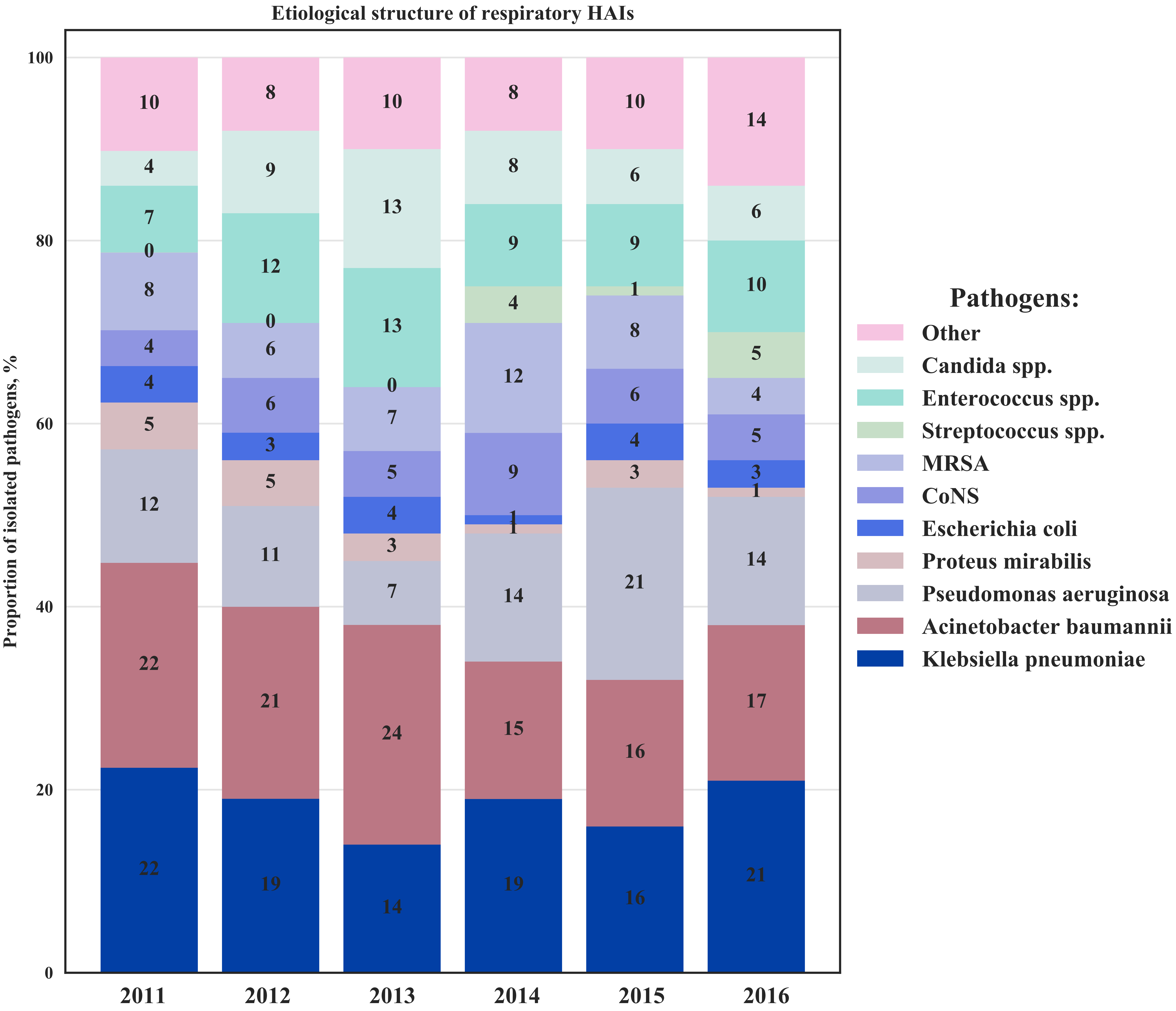

Figure 1 Supplementary. The dynamics of etiological structure of respiratory HAIs at the high-risk patient population in neuro-ICU from 2011 to 2016. Abbreviations: MRSA - methicillin-resistant *Staphylococcus aureus*; CoNS - coagulase-negative Staphylococci.

Supplementary Materials

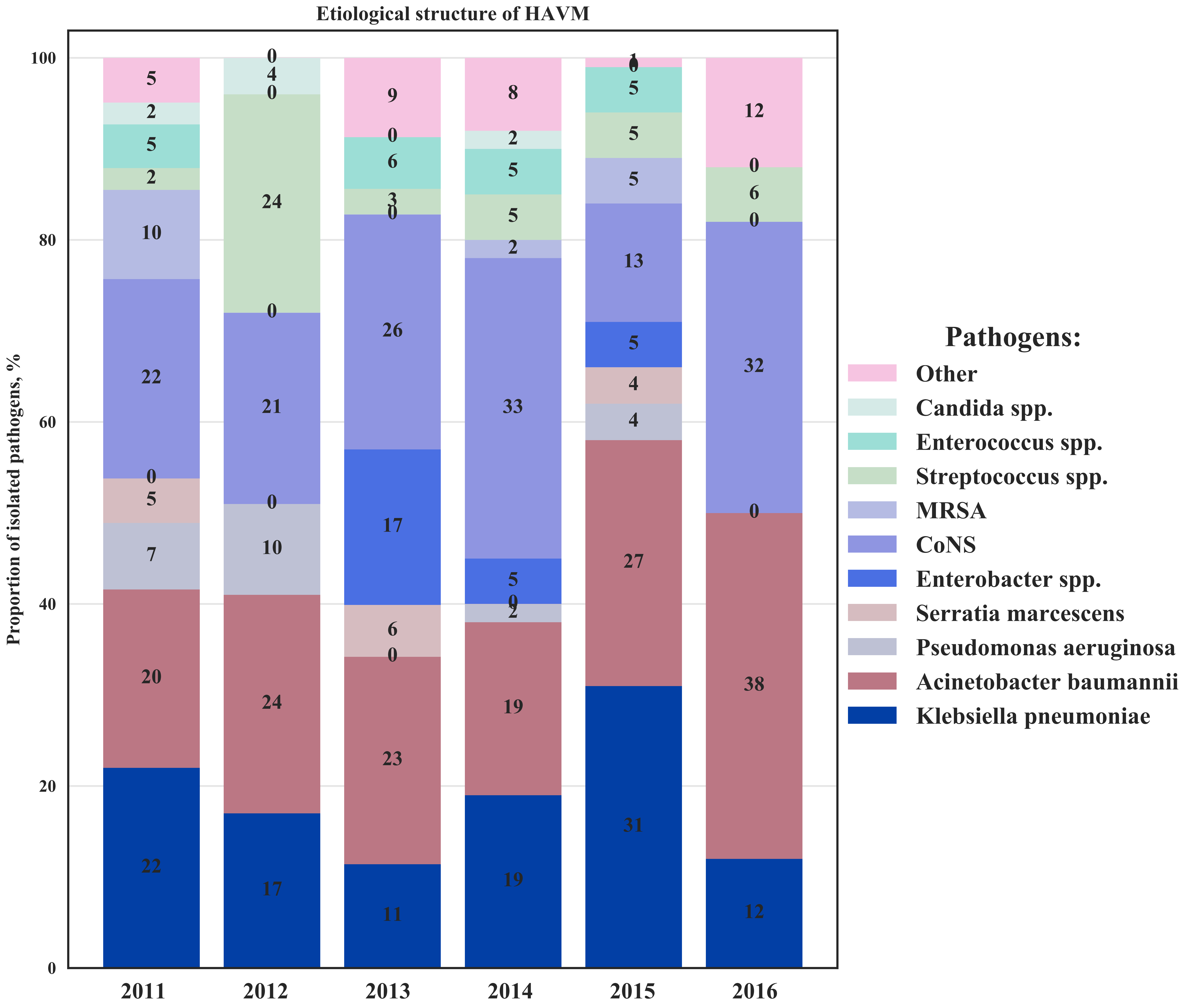

Figure 2 Supplementary. The dynamics of etiological structure of healthcare-associated ventriculitis and meningitis (HAVM) at the high-risk patient population in neuro-ICU from 2011 to 2016. Abbreviations: MRSA - methicillin-resistant *Staphylococcus aureus*; CoNS - coagulase-negative Staphylococci.

Supplementary Materials

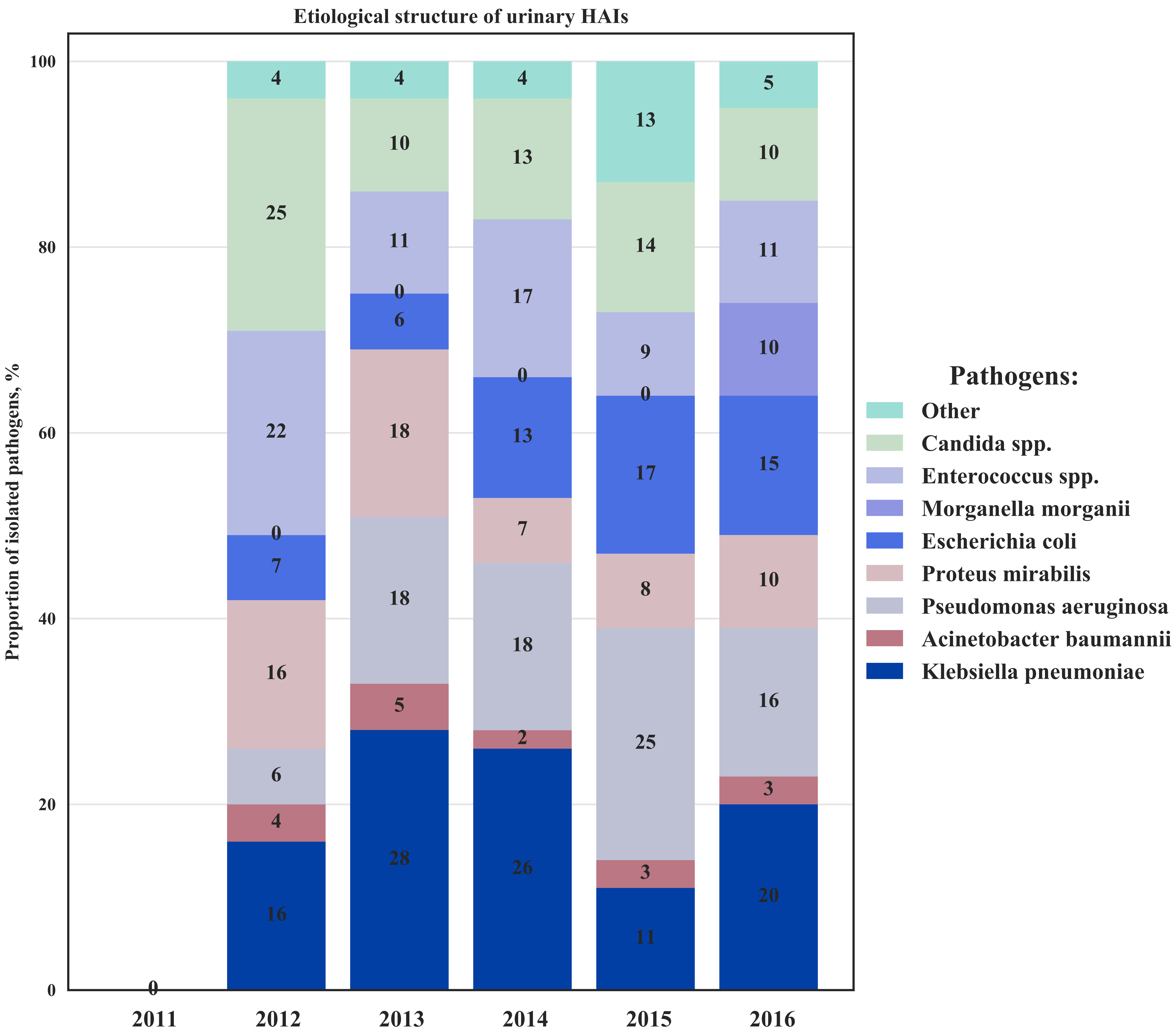

Figure 3 Supplementary. The dynamics of etiological structure of urinary tract HAIs at the high-risk patient population in neuro-ICU from 2011 to 2016. The monitoring of etiological structure of urinary tract HAIs was started in 2012.

Supplementary Materials

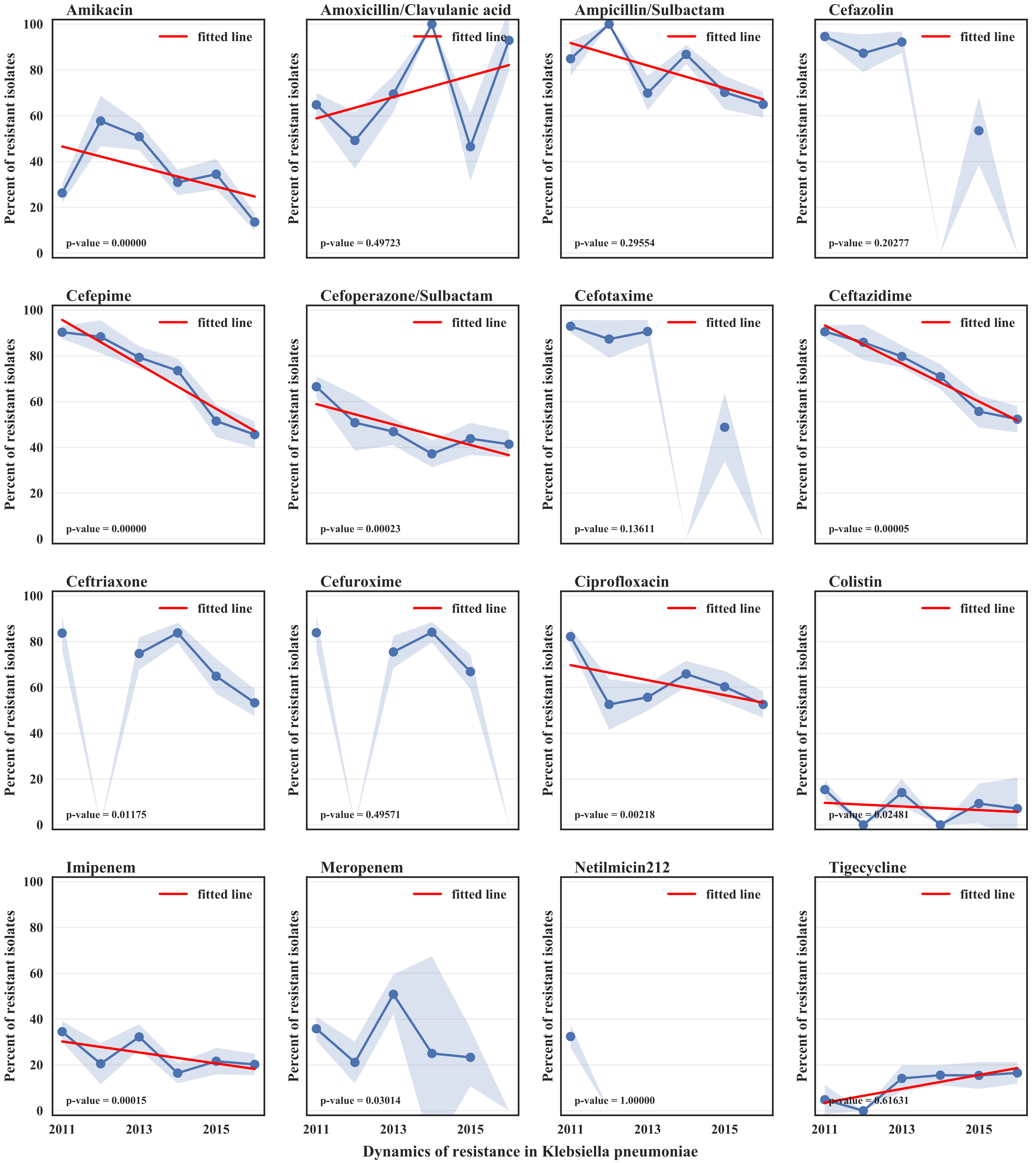

Figure 4 Supplementary. The dynamics of *Klebsiella pneumoniae* resistance to different antibiotics. Bacterial isolates were obtained from patients who had HAIs and were included in the study. Shadowed area represents 95% confidence interval for binomial distribution, p-values calculated by Chi-square test.

Supplementary Materials

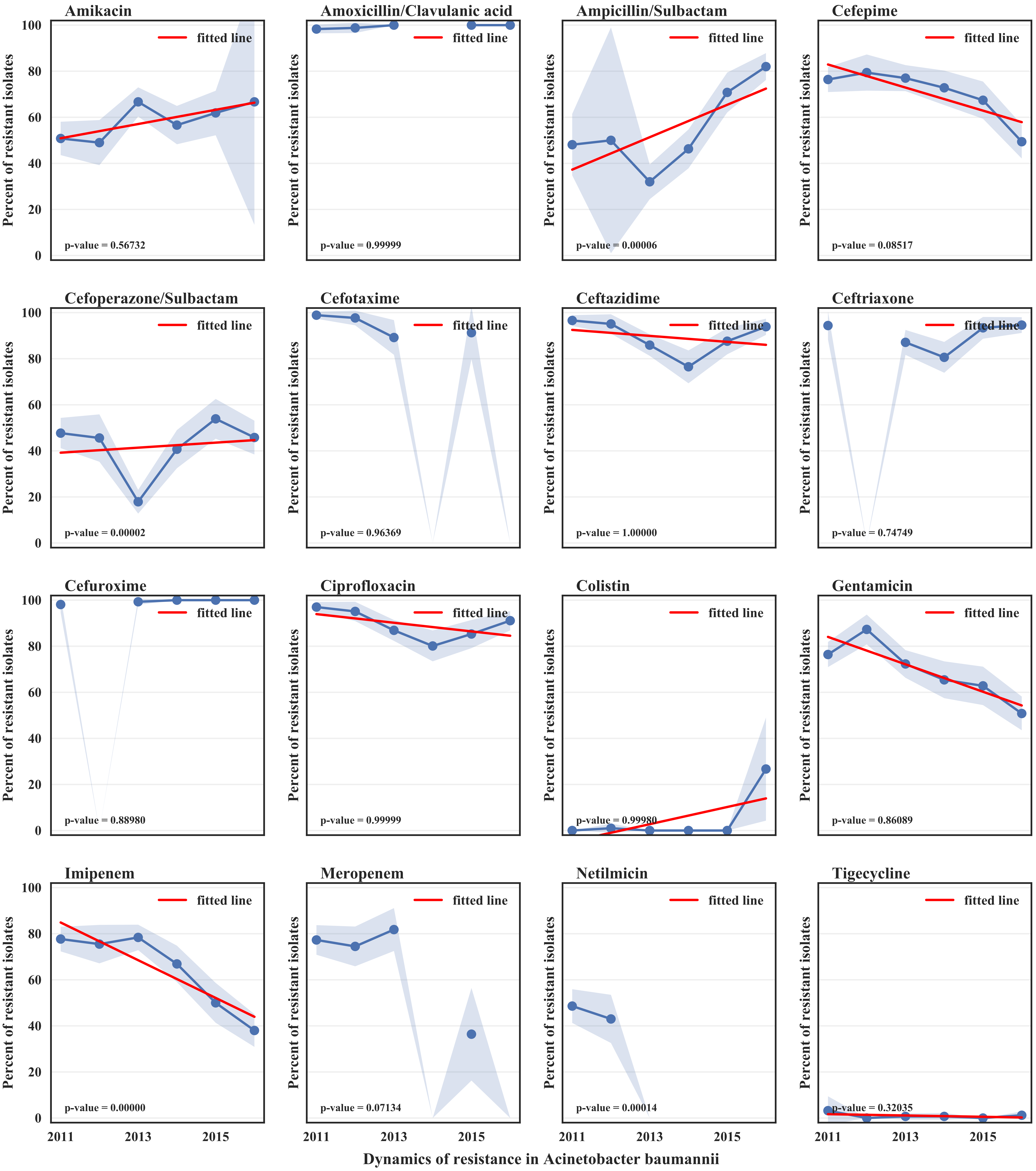

Figure 5 Supplementary. The dynamics of *Acinetobacter baumannii* resistance to different antibiotics. Bacterial isolates were obtained from patients who had HAIs and were included in the study. Shadowed area represents 95% confidence interval for binomial distribution, p-values calculated by Chi-square test.

Supplementary Materials

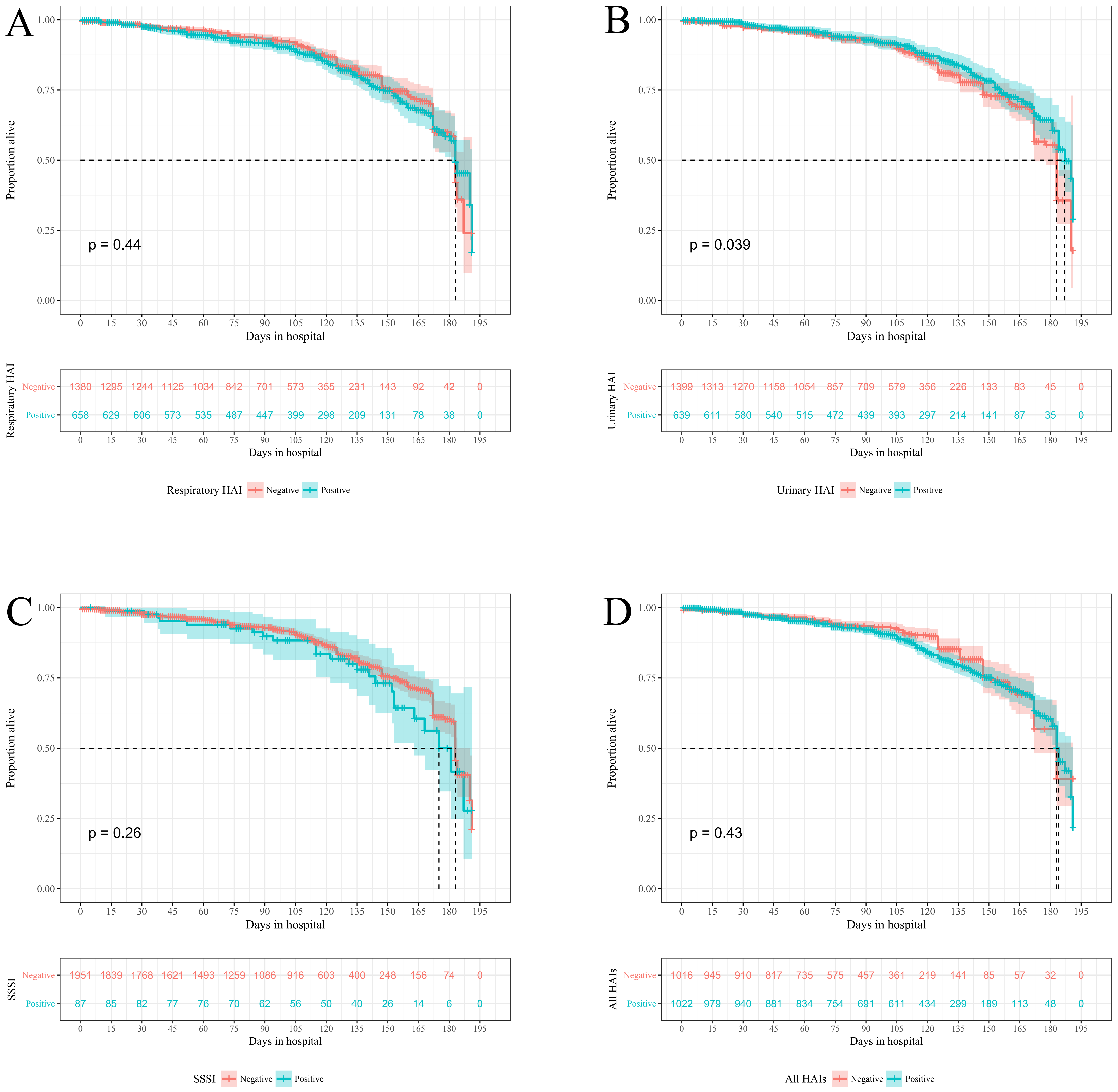

Figure 6 Supplementary. Survival curves for patients with and without different HAIs over six-year study period. Shadowed area with corresponding color represents 95% confidence interval. Number of patients at risk in each group is presented in table below the graph. **A** - respiratory HAIs; **B** - urinary tract HAIs; **C** - superficial surgical site infection after neurosurgery; **D** - patients with at least one HAI.

Supplementary Materials

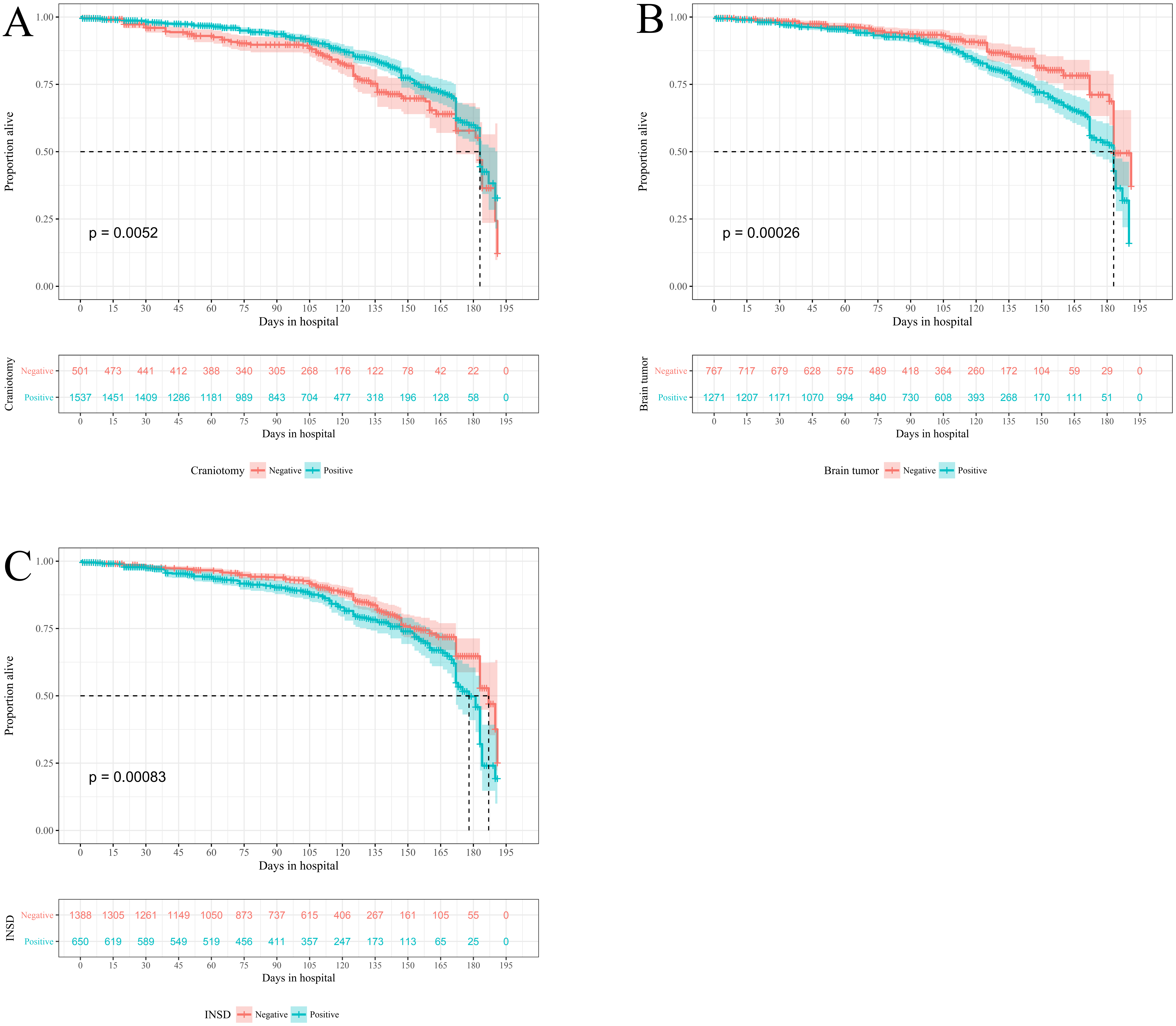

Figure 7 Supplementary. Survival curves for patients with and without different risk factor over six-year study period. Shadowed area with corresponding color represents 95% confidence interval. Number of patients at risk in each group is presented in table below the graph. **A** — craniotomy during hospital stay; **B** — diagnosis of brain tumor on admission; **C** — implantation of neurosurgical devices during hospital stay.

Supplementary Materials

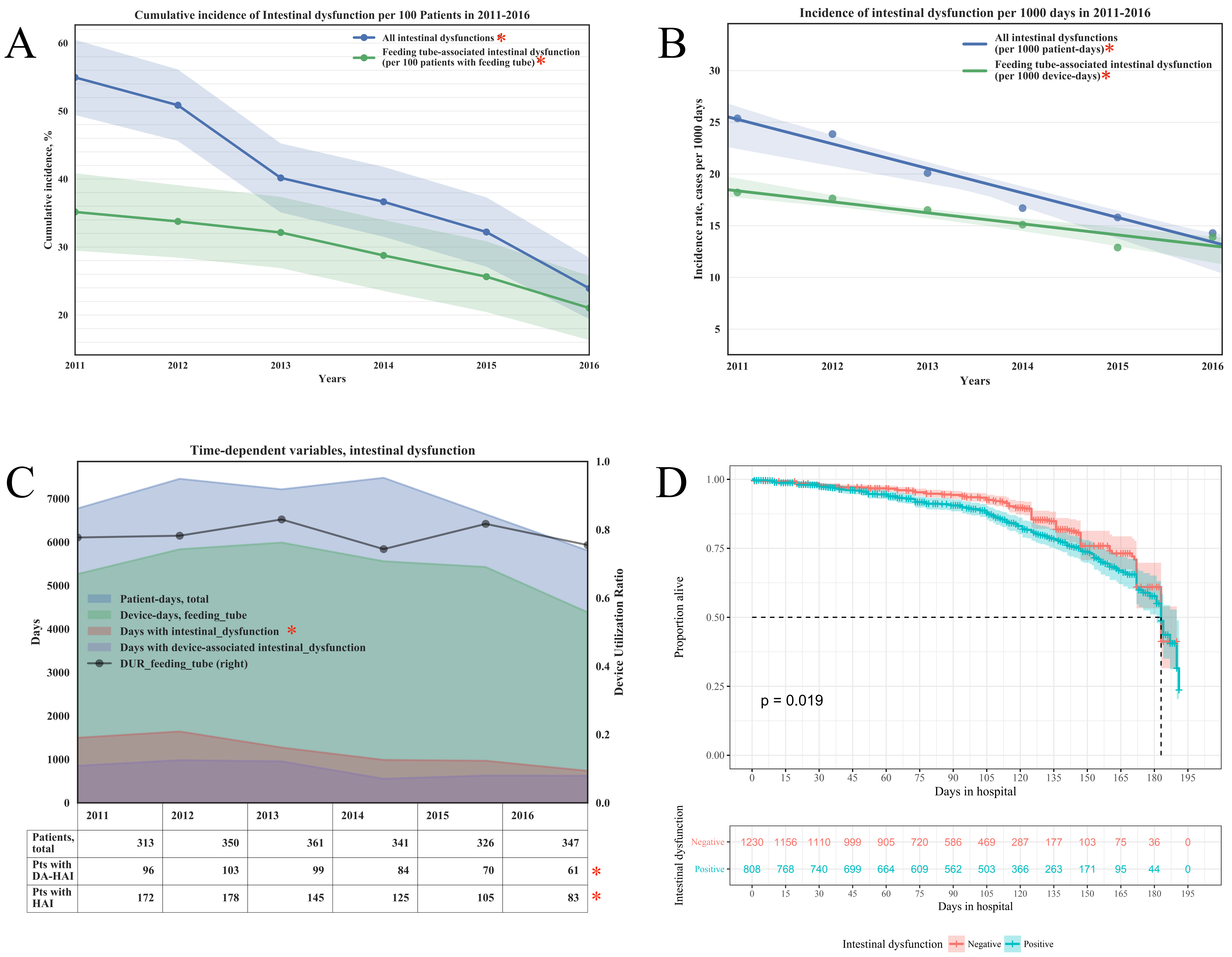

	2011	2012	2013	2014	2015	2016	
Patients, total	313	350	361	341	326	347	
Pts with DA-HAI	96	103	99	84	70	61	*
Pts with HAI	172	178	145	125	105	83	*

Figure 8 Supplementary. ICU-acquired intestinal dysfunction in high-risk ICU population in 2011-2016. **A** - cumulative incidence (%, shadowed area represents 95% confidence interval) of intestinal dysfunction. **B** - incidence rate of intestinal dysfunction per 1000 days (shadowed area represents the confidence interval for the regression estimate); **C** - time-dependent variables and device utilization ratio for feeding tube. **D** - survival curves in patients with and without intestinal dysfunction. Star (*) marks p-value < 0.05 in group comparison.
